# A pilot study on the global practice of informed consent in paediatric dentistry

**DOI:** 10.3389/froh.2024.1298277

**Published:** 2024-03-01

**Authors:** Nicoline Potgieter, Gemma Bridge, Marlies Elfrink, Morenike Oluwatoyin Folayan, Sherry S. Gao, Sonia Groisman, Ashwin Jawdekar, Arthur M. Kemoli, David Lim, Phuong Ly, Shani A. Mani, Ray Masumo, Joana Monteiro, Marjorie K. Muasya, Ambrina Qureshi, Norman Tinanoff

**Affiliations:** ^1^ Department of Orthodontics & Paediatric Dentistry, University of the Western Cape, Cape Town, South Africa; ^2^ School of Earth and Environment, The University of Leeds, Leeds, United Kingdom; ^3^ Mondzorgcentrum Nijverdal, Nijverdal, Netherlands; ^4^ Paediatric Research Project (PREP), Barneveld, Netherlands; ^5^ Department of Child Dental Health, Obafemi Awolowo University, Ile-Ife, Nigeria; ^6^ Nigerian Institute of Medical Research (NIMR), Lagos, Nigeria; ^7^ Community Oral Health Department, Tehran University of Medical Sciences, Tehran, Iran; ^8^ Faculty of Health Sciences, University of Zaragoza, Zaragoza, Spain; ^9^ Department of Stomatology, School of Medicine, Xiamen University, Xiamen, China; ^10^ Institute of Biological Sciences, DNA Diagnostic Laboratory, University Stadual of Rio de Janeiro, Rio de Janeiro, Brazil; ^11^ Department of Paediatric and Preventive Dentistry, Bharati Vidyapeeth Deemed to be University Dental College and Hospital, Navi Mumbai, India; ^12^ Department of Paediatric Dentistry and Orthodontics, University of Nairobi, Nairobi, Kenya; ^13^ Tzu Chi Free Clinic, Buddhist Compassion Relief Tzu Chi Foundation Singapore, Singapore, Singapore; ^14^ Geriatric and Special Care Dental Clinic, National Dental Centre, Singapore, Singapore; ^15^ Special Oral Care Network, Singapore, Singapore; ^16^ Department of Development Studies, The Graduate Institute Geneva, Geneva, Switzerland; ^17^ Department of Paediatric Dentistry & Orthodontics, Faculty of Dentistry, Universiti Malaya, Kuala Lumpur, Malaysia; ^18^ Department of Community Health and Nutrition, Tanzania Food and Nutrition Centre, Dar es Salaam, Tanzania; ^19^ Department of Paediatric Dentistry, Sheffield Teaching Hospitals, Sheffield, United Kingdom; ^20^ Community Dentistry, Dow University of Health Sciences, Karachi, Pakistan; ^21^ Department of Orthodontics and Pediatric Dentistry, School of Dentistry, University of Maryland, Baltimore, MD, United States

**Keywords:** informed consent, child consent, self-consent, ethical practice, medical consent, paediatric dentistry

## Abstract

**Background:**

Conducting oral treatment early in the disease course, is encouraged for better health outcomes. Obtaining informed consent is an essential part of medical practice, protecting the legal rights of patients and guiding the ethical practice of medicine. In practice, consent means different things in different contexts. Silver Diamine Fluoride (SDF) and Silver Fluoride (SF) is becoming popular and cost effective methods to manage carious lesions, however, cause black discolouration of lesions treated. Obtaining informed consent and assent is crucial for any dental treatment—and has specific relevance with SDF/ SF treatments.

**Methods:**

The aim of this paper is to describe informed consent regulations for dental care in a selection of countries, focusing on children and patients with special health care needs. An online survey was shared with a convenience sample of dental professionals from 13 countries. The information was explored and the processes of consent were compared.

**Results:**

Findings suggest that there are variations in terms of informed consent for medical practice. In Tanzania, South Africa, India, Kenya, Malaysia and Brazil age is the determining factor for competence and the ability to give self-consent. In other countries, other factors are considered alongside age. For example, in Singapore, the United Kingdom, and the United States the principle of Gillick Competence is applied. Many countries' laws and regulations do not specify when a dentist may overrule general consent to act in the “best interest” of the patient.

**Conclusion:**

It is recommended that it is clarified globally when a dentist may act in the “best interest” of the patient, and that guidance is produced to indicate what constitutes a dental emergency. The insights gathered provide insights on international practice of obtaining informed consent and to identify areas for change, to more efficient and ethical treatment for children and patients with special needs. A larger follow up study is recommended to include more or all countries.

## 1 Introduction

Oral diseases pose a major health burden, affecting over 3.5 billion people globally (1). According to the Global Burden of Disease survey 2017, untreated dental caries in permanent teeth is the most common health condition, whilst over 530 million children suffer from dental caries of primary teeth annually (2). If left untreated, dental caries can lead to pain, tooth loss and infection, affecting quality of life and productivity. As such, conducting treatment early in the disease course is encouraged.

In addition to dental caries, children also suffer from traumatic dental injuries, which are very common in the first ten years of life. These injuries are more common particularly in school-going children who may be involved in sports activities. These injuries may include not only laceration of chin and lips but also fractures of facial bones and teeth that require children to be taken to a dentist for further management and treatment (3).

Obtaining informed consent before treatment, delivered to a patient is an essential part of contemporary medical practice, protecting the legal rights of patients and guiding the ethical practice of medicine (4). Informed consent is when a person gives permission before they receive treatment or an examination after receiving all pertinent information about the procedure. In practice however, consent means different things in different contexts, largely determined by whether consent is being obtained for legal, ethical or administrative purposes.

Globally, persons who can provide consent varies. Typically, whether or not a patient can provide consent themselves is determined by each patient's comprehension of the treatment proposed (5). Younger children, Patients with Special Health Care Needs (PSHCN) or those who are incapacitated, and are unable to fully understand the treatment process or purpose, require consent for care provided by legal parents, guardians or an acting caregiver. In such situations, assent for treatment by children or PSHCN is promoted. Assent pertains to actively involving the patient in making and taking decisions about their own body and health (6).

The aim of this article is to describe informed consent regulations for dental care in a selection of countries, focussing on children and PSHCN. This paper's view of consent aims to provide insights on international practice of obtaining informed consent for paediatric dentistry and to identify areas for change, to more efficient and ethical treatment of children and PSHCN populations.

## 2 Methods

The cross-sectional study was conducted, in 2021/22, by means of a self-administered questionnaire, by representatives from different countries. The selection of countries was based on personal contacts and past research collaborations. The questionnaire asked each representative to provide information on the policies, guidelines and practice of informed consent and assent in the respective countries/areas. A convenience sample of 13 countries/jurisdictions across the globe were used. The data collected through the questionnaire. The principle researchers collated the data from the questionnaires and and cross-checked the data with the laws and regulations referenced. Once all data were combined, tabled and organized, all authors contributed to the discussion to bring context of the different countries' laws and regulations.

## 3 Results

### 3.1 Different measures of competency enable self-consent

Globally, parent or legal guardian are required to give informed consent to “minors” for medical/dental treatment. Age is the main determinant of being a minor in most countries. Table 1 summarizes the legal ages for obtaining consent for dental treatment for children and exceptions to the “age-rule”. Of the countries included in this evaluation, Tanzania, South Africa, India, Kenya, Malaysia and Brazil fully rely on age as determining factor for competence and the ability to give self-consent: In contrast, Pakistan and Singapore do not have a standard age for self-consent to medical/dental treatment and instead, only require verbal consent from parents/guardians or family members for treatment (23, 26).

**Table 1:** Summary of the legal requirements for consent and the age of self-consent for dental treatment.

	Age of giving self-consent	Exceptions to enable giving self-consent as a minor	Act/law/policy stipulating specific age for self-consent	Summary of consent allowed during an emergency situation	Assent legally required?
Brazil (7–9)	>18 years	None	No 2-SEI/2017-CGSAJ/DAPES/SAS/MS, together with Children and Teenager Statute, and The Brazilian Dental Ethical Conduct (Resolution CFO—118/2012) in the article 11°	– Medical professionals are forbidden from performing any medical procedure without previous explanation and consent of the patient or his/her legal representative, except in cases of imminent threat to life	No
China (10)	≥18 years	<8 years has no civil capacity and 8–17 years restricted civil capacity	Civil Code of People's Republic of China Chapter 2 and Chapter 6	– Dentists may provide treatment with the approval by the person-in-charge of the hospital	No
India (11–15)	>18 years	12–18 years can consent for examination but not procedures	Majority Act, Guardian and Wards Act, and Indian Contract Act	– “Loco parentis” person in charge: example: Principle of the school. – No consent needed in medical emergency/ life endangerment	No
Kenya (16, 17)	>18 years	None	Kenyan Children Act No 8 of 2001	– Reasonable steps to obtain consent – Clinician – Court of law – Next of kin – Not necessary in emergency that could result in death or irreversible damage	No
Malaysia (18, 19)	>18 years	None	The Laws of Malaysia Act 21: Age of Majority Act 1971 and Malaysian Medical Council guideline: Consent for treatment of patients by registered medical practitioners	– Court of law – “Protector” when child is in protective custody – When parent or legal guardian is unavailable, Primary and secondary physician co-sign during life endangerment – Dental practitioner may act for the best interest of the patient, in consultation with parents or legal guardian	No
Maryland state USA (20)	>18 years	Gillick Competency^a^	People's Law Library of Maryland	– Minor has the same capacity as an adult	No
Netherlands (21)	>16	Gillick Competency^a^	Wet Geneeskundige BehandelOvereenkomst	– Dentists may act to create a non-acute situation.	Verbally Assent
Nigeria (22, 39)	≥18 years	Married woman and head of households can give consent regardless of their age.	Section 29 (4) of the 1999 Constitution of the Federal Republic of Nigeria	– Dentist to avoid death or serious harm – Police – Nigerian Supreme Court	Yes
Pakistan (23–25)	None set for routine dental procedures. Self-consent is required only major surgical procedures ≥ 18 years	Gillick competence^a^	Majority Act, 1975	– Physician – Consultation with family members	No
Singapore (26–29)	>21 years	Gillick competence^a^	No statute law, however “…p*resumption in law and medical practice is that all adults have capacity to consent or refuse treatment, unless proven otherwise…*”	– Doctor/dentist acting in “best interest” – Some healthcare institutions may necessitate a “double clinician consent”, where a registrar-level and a consultant-level doctor/dentist will discuss and concur on a shared best-interest decision. This is not required legally, but an organisational policy.	No
South Africa (6)	≥12 years	With sufficient maturity Need accompanied parental consent for surgical procedures <16 years	Children's Act No 38 of 2005	– Court appointed Social worker – Clinical manager/Superintendent of hospital	Yes
Tanzania (30)	>18 years	None	The Law of the Child Act, No.21 of 2009	– Government authorities – Doctor/Dentist – Police	Yes
United Kingdom (31, 32)	≥16 years	Gillick Competency^a^	The Children Act 1995 The Children Act 1995 (England, Wales, Scotland) The Children Order 1995 (Northern Ireland)	– Any adult with parental responsibility. – Practitioner in the “best interest of a child”	No
Vietnam (33–37)	>18 years	15–18 years may perform civil transactions (this may be argued as dental treatment)	None	– Legal representative or representative appointed by court – Head of medical facility – Dentist/medical practitioner	No

^a^ Gillick Competency: is used to identify children aged under 16 who have the legal competence to consent to medical treatment. Competency is conferred if the child can demonstrate sufficient maturity and intelligence to fully understand the proposed treatment and its implications, including an understanding of the risks and any possible alternative course of action (38).

In other countries, there are varying exceptions and multiple factors that are taken into consideration to apply the “age-rule” for consent. In Singapore, the United Kingdom, and the United States the principle of Gillick Competence is applied. Gillick Competence is used to identify children aged under 16 who have the legal competence to consent to medical treatment. Competency is conferred if the child can demonstrate sufficient maturity and intelligence to fully understand the proposed treatment and its implications, including an understanding of the risks and any possible alternative course of action (38).

The Netherlands also have an age range (12–16 years) wherein both the parent and the child should give consent with two exceptions: (1) if not treating the child will give a serious disadvantage; (2) If the treatment is the well-considered wish of the child. In Nigeria, the social status of the child's family can influence the decision to give self-consent (21). If a child, regardless of their age, is married, they are considered as an emancipated minor, which means that they can give consent. Likewise, if a child becomes the head of a household, for example as a result of both parents dying, then again, they are considered capable of providing consent for medical treatment.

In Vietnam, for those who are from 15 to 18 years old, the civil act capacity determines the ability of the individual to provide consent. The civil act capacity of an individual is his or her capability to establish and perform civil rights and obligations. Per article 21 of the Civil Code, those who are above six years of age also have civil act capacity. However, in a similar vein to the Gillick Competency test, if the patient is incapable of “recognizing or controlling his/her acts due to mental disease or other ailments”, the court may revoke or restrict their civil act capacity (article 22 and 23). In such a case, a legal representative will give consent to medical/dental treatments on their behalf (35).

Like Vietnam, India applies age as a determinant of competence, however comprehension of treatment is also considered, whereby an incompetent patient is one “who is a minor” or a person of unsound mind or a patient who is unable to weigh, understand or retain the relevant information about his or her medical treatment; unable to make an informed decision because of impairment of or a disturbance in the functioning of the mind or brain: or a person who is unable to communicate the informed decision regarding medical treatment through speech, sign or language or any other mode (40).

### 3.2 Parents/legal guardian refusing consent

When patients are not deemed capable of providing consent themselves, it is requested that a parent or legal guardian consent on their behalf. However, in some situations, one or two of the parents or guardians may refuse to provide consent for treatment. In this situation, there are different procedures in place depending on the context.

In India, “when the parents/guardian refuses their child to undergo the diagnostic procedure/treatment after a complete and comprehensive information has been provided, the parents should be informed in a discreet professional manner of consequences of refusal, failing that the physician can be held liable in the court of law. The conflict of “best interest standards” for treatment of the child vs. “rational parent standard” for the attitude of parents is matter of never ending debate” (11). In Malaysia, when one parent consents and the other refuses, counselling of the parents to reach an agreement on what is in the child's best interest is conducted. A decision can be made on a case-by-case basis in the best interest of the patient by the medical practitioners when the patient is in a situation of helplessness and the decision is the most appropriate and fair to that child under the circumstances. If the child is under the temporary custody, the protector or police officer may authorise treatment without obtaining consent from the parent/guardian in certain circumstances.

In Tanzania, Section 4 (2) of the Law of the Child Act insists that the best interest of a child should be the primary consideration in all actions pertaining to the child. Also Section 9 (1) of the same Act provides for the right to life, dignity, respect, leisure, liberty and health for children and parents, guardians and relatives are responsible to ensure the enjoyment of those rights. Again Section 13 (1) of the same Act prohibits degrading treatments to children, and institutions like local government authorities and police are vested with legal duties under the said Act to ensure children's safety (30). Similarly, in *Singapore*, the healthcare provider is expected to uphold the “best interest” of the patient or child, in line with the Mental Capacity Act, regardless of who holds the ultimate authority in healthcare decision making (29). Should a decision, or non-decision (willful neglect or acts of omission), lead to potential harm of the child, this would constitute a child protection concern and other actors are brought in to resolve the conflict. Initially this is sought from a single or multiple agencies, such as a school. If resolution is not sought, then the Child Protection Service (CPS) is brought in (40).

In the United Kingdom, as in Singapore, another actor is brought in if the young person themselves, or the individual with parental responsibility refuses to give consent to medical treatment for the young person or child. In most countries participating in this study, the Court of Protection is brought in and can overrule the decision if it is deemed to be in the best interest of the young person or child, such as, if refusing the treatment may result in the death or permanent injury.

### 3.3 Country specific ethical dilemmas

There are several country-specific issues related to consent that add to the complexities of securing informed consent for dental treatments.

In India, most dentists are unaware of all the necessary aspects of the consent process (41, 42). This could be explained by the lack of guidance in how to obtain consent, in the Dental Council of India's Code of Ethics (43). In a similar vein, according to a recent study in Pakistan, it was found that 93% of parents are not aware that they need to obtain a copy of a signed informed consent document for their record (23). This reflects the lack of knowledge within the country related to informed consent and rights.

In Nigeria, an ethical dilemma dentist's face is the request to extract the maxillary central primary incisors when they erupt ahead of the lower central incisors. This is because of the widespread belief that a child who erupts the maxillary central primary incisors ahead of the lower has supernatural abilities that makes the child able to pronounce curses. Parents therefore, request dentists to remove these teeth otherwise they hide the child out of sight from guests until the lower teeth fully erupts or get “quacks” to do the extraction. The presence of natal/neonatal teeth gives similar concerns to the parents. Also, a diastema is regarded as a sign of beauty and patients sometimes visit the dentist to create an artificial diastemas (44). Dentists are faced with ethical challenges when the size of the requested diastema is large with increased risk for exposure of dentine and its attendant complications.

In Singapore, healthcare decision making can be affected by the source of financing. Singapore's healthcare utilises a mixed financing system (45). Particularly for dental services, where limited subsidies are available, procedures that are arguably elective, such as orofacial reconstruction or orthodontic cases, may be costly. For example, a child may wish to retain a primary molar by pulpotomy and stainless steel crown, while the parents may deem this too expensive and opt instead for an extraction. As such, treatment decision can become intertwined with willingness-to-pay (46). Patients also face this dilemma in Nigeria.

In Vietnam, an ethical dilemmas often arises when family members of the child disagree on an emergency treatment. Due to the Law on Medical Examination and Treatment, physicians are legally prevented from delivering care, unless the patients' family comes to a consensus (47). However, there is a new draft of the Law on Medical Examination and Treatment, submitted to the National Assembly in March 2021, which proposed that a doctor or dentist provide treatment to a child, even if the parents object to giving consent for treatment on behalf of the child (48).

If the Gillick Competency test is met, then the child is deemed capable of providing consent to treatment (38). In countries applying Gillick competency, ethical dilemmas arise when children who are considered Gillick competent choose different treatment options than the parents. If a Gillick competent child gives consent this may not be overruled by the parents if the practitioner feels the treatment is appropriate.

Beyond the country specific issues related to obtaining informed consent highlighted above, there are also cultural or religious practices that, due to their risk of harm to patients, they raise ethical concerns and have specific consent related considerations. In Pakistan, the religious beliefs can be problematic as some believe a negative medical outcome is God's Will, and as a result they would prefer to accept their child's fate and pray, rather than to provide consent for treatment (49). In Tanzania, due to religious or cultural beliefs, some parents enucleate the tooth buds of as a way of reduction the risk for infections and illnesses (in Swahili- Dawa ya Jino Kung'oa), contrary to the recommendation of dentists or dental professional boards. Consent is often not sought from the parent nor assent from the child (in case of lower incisor extraction). Similar practices are also seen in several other East African countries such as Kenya (50). In South Africa, dentists are sometimes faced with the ethical dilemma of children, over the legal age of consent (12 years), requesting their four maxillary incisors to be extracted with the parents' approval, as it is believed that it will make the individual more “attractive and romantic” and will therefore ensure a better life partner (51).

## 4 Discussion

Dentists, as professional health care providers, are required to provide care based on sound ethical principles. Ethics is defined as “the moral principles or virtues that govern the character and conduct of an individual or a group” (52). Ethics therefore refers to our actions and how we relate to one another as human beings. It includes our intentions and the consequences, even when one's actions don't always result in the intended outcome. In more simple terms we can translate ethics as: do unto others as you would have them do unto you. However, acting ethical is most often more complex than definitions or principles and the challenges faced in clinical situations. Although ethical principles and guidelines are available to guide dental practitioners—these principles are subject to different individual interpretations. Nonetheless, ethics forms the core of all decision making, patient relationships, hence being an ethical professional and is a lifelong process through consistent ethical behaviour.

The ethical principle “Autonomy” refers to “Patients have the right to determine what should be done to their bodies” (52) and the parents or legal guardians have the right to decide what happens to their children's bodies. In simple terms autonomy can be described as informed consent: “permission granted in full knowledge of the possible consequences, typically that which is given by a patient to a doctor for treatment with knowledge of the possible risks and benefits” (53). The process must be explained to the patient requiring any treatment/clinical intervention, and involving that patient in decision making; and even if that involvement is limited, it is critical in meeting the legal, ethical and administrative requirements of informed consent. However, enabling true informed consent can be challenging and requires flexibility, especially where young children and PSCHNs are concerned.

Although all countries included in this study have governing, rules and regulations regarding consent and the age of consent, the final decision making falls to the dentist. “Regardless of the parent's request, the dentists’ primary ethical, moral and legal duty is to the child” (54). Even the Gillick Competent principle assumes that “the child has the maturity and ability to understand what the treatment involve as determined by medical practitioner, i.e., they have been assessed to have adequate capacity (i.e., intelligence, competence and understanding) to be able to make an informed decision about their treatment”. Although subjective, the dental practitioner responsible to determine if the child is Gillick competent or not.

Many dental practitioners would like to apply the “best interest” principle in routine practice, thereby taking control of the decision making regarding treatment of minors. However, the responses from most countries confirmed that dentists may only act in the best interest of the patient and treat the patient without formal consent if it is a life threatening situation and/or there is no legal person available to give consent. However, in Nigeria children are primarily considered the property of the State and parents are custodians of children on behalf of the State (55). This rights-oriented model for child care limits absolute parental control over the child and gives the State the right to interfere in the care of the child (44). The Health Professions Council of South African guidelines states that the “best interest” principle does not apply to patients who are competent, have sufficient maturity and mental capacity (53). With the exception of Pakistan, dental practitioners globally are dependent on consent for dental treatment obtained by a legal parent/guardian or the child of age for routine dental care. Informed consent therefore requires a dynamic interaction between the dentist, patient and parent/legal guardian. Dentist must provide sufficient information and education to enable parents and children who can self-consent to make the right decisions.

Although some countries' laws mention the dentists' authority to act in emergency situations, what constitutes an emergency in the dental setting is not specified or defined. Most laws only refer to “emergency” or “life threatening” situations, which leaves room for individual interpretation regarding dental conditions. Examples of dental emergencies are trauma, abscess formation (without facial swelling) or a lost filling among other. However, it is debatable if these dental conditions can be considered as “emergencies” for which dentist may overrule consent/assent or even act in the best interest of the child regardless of consent.

A limitation of this study is that it only includes 13 countries and a larger follow up study involving more or all countries should be done. The results of this pilot study can therefore not be safely generalized.

## 5 Conclusion

The policies and guidelines enabling children to self-consent for dental treatment varies globally. Some countries make use of a combination of age and Gillick Competency, whereas others strictly adhere to age as determining factor for competency. Obtaining assent from a child-patient is compulsory in some countries, but not specified in laws or regulations in all countries. Many countries' laws and regulations do not specify when a dentist may act in the “best interest” of the child- overruling general consent rules. Taking into consideration the aesthetic implications of SDF/SF treatments, obtaining informed consent plays a crucial role in successful management of caries with these products.

It is recommended at three areas are clarified to help dentists in providing appropriate dental care; first, when a dentist may act in the “best interest” of the child. Second, guidance what constitutes a dental emergency and third, clarity within the law with regard to overruling consent. A larger follow up study is recommended to include more or all countries around the world.

## Data Availability

The original contributions presented in the study are included in the article/supplementary material, further inquiries can be directed to the corresponding author.
